# De-Aliasing and Accelerated Sparse Magnetic Resonance Image Reconstruction Using Fully Dense CNN with Attention Gates

**DOI:** 10.3390/bioengineering10010022

**Published:** 2022-12-22

**Authors:** Md. Biddut Hossain, Ki-Chul Kwon, Shariar Md Imtiaz, Oh-Seung Nam, Seok-Hee Jeon, Nam Kim

**Affiliations:** 1 School of Information and Communication Engineering, Chungbuk National University, Cheongju-si 28644, Chungcheongbuk-do, Republic of Korea; 2 Department of Electronics Engineering, Incheon National University, 119 Academy-ro, Yeonsu-gu, Incheon 22012, Gyeonggi-do, Republic of Korea

**Keywords:** aliasing artifacts, attention gate, deep learning, fully dense network, MRI reconstruction

## Abstract

When sparsely sampled data are used to accelerate magnetic resonance imaging (MRI), conventional reconstruction approaches produce significant artifacts that obscure the content of the image. To remove aliasing artifacts, we propose an advanced convolutional neural network (CNN) called fully dense attention CNN (FDA-CNN). We updated the Unet model with the fully dense connectivity and attention mechanism for MRI reconstruction. The main benefit of FDA-CNN is that an attention gate in each decoder layer increases the learning process by focusing on the relevant image features and provides a better generalization of the network by reducing irrelevant activations. Moreover, densely interconnected convolutional layers reuse the feature maps and prevent the vanishing gradient problem. Additionally, we also implement a new, proficient under-sampling pattern in the phase direction that takes low and high frequencies from the k-space both randomly and non-randomly. The performance of FDA-CNN was evaluated quantitatively and qualitatively with three different sub-sampling masks and datasets. Compared with five current deep learning-based and two compressed sensing MRI reconstruction techniques, the proposed method performed better as it reconstructed smoother and brighter images. Furthermore, FDA-CNN improved the mean PSNR by 2 dB, SSIM by 0.35, and VIFP by 0.37 compared with Unet for the acceleration factor of 5.

## 1. Introduction

Magnetic resonance imaging (MRI) is a sophisticated and noninvasive medical imaging technique that has full control over data capture to visualize the anatomy and functions of the human brain and body [1]. It plays a significant role in smart healthcare systems alongside medical research by providing high-quality reconstructed images without exposure to harmful radiation [2]. However, the long image acquisition time (more than 30 min per patient) [3] is a disadvantage compared with X-ray, computed tomography (CT), and photoacoustic tomography (PAT) imaging modalities. In MRI, frequencies are acquired in the k-space rather than directly in the image space. Full k-space data are required for high-quality reconstructed images, but this prolongs the acquisition process. This slow process causes the patient discomfort and generates motion artifacts due to patient movement. A common way to speed up MRI acquisition is by taking fewer scans and reconstructing the image using a partially recorded k-space. However, this creates blurring and aliasing artifacts in the images according to the Nyquist–Shannon principle [4]. Hence, an effective acceleration technique is essential for MRI reconstruction. Parallel imaging (PI) [5,6] applies coil sensitivity maps to speed up reconstruction, but the setup process is complex and expensive. Compressed sensing (CS) MRI [7,8] uses sparse data to reconstruct high-resolution images from randomly picked k-space data. However, the sparsity of data produces aliasing artifacts, and the computing time required is quite high for their iteration process.

Deep learning (DL) has been successfully adopted in medical image processing [9,10,11,12] and deals with the obstacles of iterative CS reconstruction [13,14]. Efficient reconstruction can be performed without repeatedly processing after properly training a DL model. Several post-processing strategies [15,16,17,18] based on generative adversarial networks (GANs) have been used to improve image quality in the image domain, whereas some approaches [19,20,21,22] estimate the unknown missing k-space frequencies in the sensor domain. Cross-domain techniques [23,24,25,26,27,28,29] operate in both the sensor and image domains. Recently, iterative unrolled optimization methods [30,31,32,33,34] have been used for sparse MRI reconstruction to improve the learning of image features. However, when the k-space is uniformly under-sampled, DL-based iterative methods cannot properly eliminate aliasing artifacts.

The first multilayer perceptron [35] is applied to reduce aliasing artifacts in parallel MRI. The multi-scale Unet structure [36] has been suggested to handle phase image reconstruction and scattered artifacts. The convolutional neural network (CNN)-based two-dimensional (2D) phase-contrast MRI reconstruction method [37] encodes low-frequency regions in the phase direction, while high-frequency regions store image edges. The GAN-based de-aliasing method [38] combines adversarial and innovative content loss but calculates fast Fourier transforms (FFTs) of magnitude images instead of raw MRI data. RefineGAN [39] measures only the cyclic loss of the training dataset using an autoencoder. Based on deep residual learning (DLR), the denoising technique [40] identifies and then subtracts noise from noisy images. After modifying this DLR-based network, Ouchi et al. [41] proposed a model to generate alias-free images by subtracting the predicted alias components from them.

Here, we adopt a post-processing strategy and propose an efficient fully dense attention convolutional neural network (FDA-CNN) architecture to remove aliasing and ghost artifacts from sparsely reconstructed 2D MRI images. The FDA-CNN method generates aliasing-free images instead of prediction artifacts, which is a key difference from previous MRI de-aliasing techniques. Guan et al. [42] proposed fully dense Unet (FD-Unet) for reducing artifacts from PAT images. We use the modified FD-Unet architecture for MRI reconstruction by adding an attention gate (AG) to each decoder layer. FDA-CNN consists of an AG [43] in each layer of the decoding path and dense connectivity (DC) [44] in both the encoding and decoding layers. DC solves the vanishing gradient issue and improves information flow, whereas the AG gives more attention to essential features that make the network more generalized. Supplementarily, we implement a hybrid mixed center and periphery under-sampling (MCP-US) mask in the phase [ reconstructs complex image textures and contents better than the two-dimensional (2D) and one-dimensional (1D) Gaussian under-sampling patterns. The results of FDA-CNN were evaluated using three sub-sampling masks using only 20% of the data. The simulation results were compared to both the single- and multidomain networks along with traditional CS methods on three widely used k-space datasets.

## 2. Methodology

The data obtained from an MRI scanner are known as the Fourier space or k-space and can be represented as follows:

(1) y|=|FT

where T is the artifact-free image, F is the Fourier transform, and y represents the fully sampled k-space data. The reconstruction of image $\hat{x}$ from the k-space can be performed by applying the inverse Fourier transform.

(2) $$\hat{x}|=|F^{–1}\left(y\right)$$

The reconstruction of an image with n pixels requires at least n frequencies. However, it takes more scan time to capture n frequencies, because the number of frequencies acquired is linearly related to the length of the MRI scan. Acquiring only 1/5 of the frequencies can reduce both the scan time and cost by a factor of 5, but aliasing artifacts are introduced. Under-sampled k-space $\hat{y}$ is represented as

(3) $$\hat{y}|=|U\;⊙\;y|=|U\;⊙\;\mathrm{FT}$$

**Figure 1:**
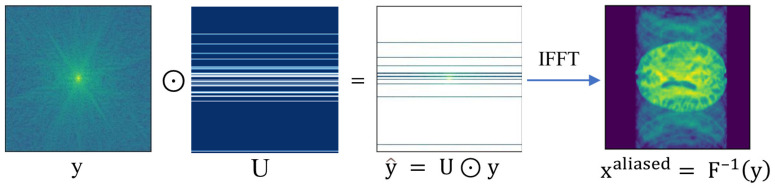
Reconstructed artifact image from sparsely sampled k-space.

Figure 1 exhibits a reconstructed artifact $x^{\mathrm{aliased}}$ image from the under-sampled k-space. Here, U is a sub-sampling matrix filled with 0 s (black area) and 1 s (white line), and ⊙ expresses the element-wise multiplication.

Conventional CS methods recover images by solving the following optimization problem:

(4)  Tmin|y^–y|22+λGT

where $\left|\right|{.||}_{2}\;$ is a generic data consistency term that assures the solution of the original image (T) with every observation of y and G denotes the regularization term, where λ expresses the regularization parameter. The challenge of traditional CS is that the regularizer, G(T), must be manually encoded to represent the reconstructed MR images.

### 2.1. Deep Learning Framework

A DL-based method is applied to adjust the under-sampled image and reconstruct an artifact-free image that is close to the actual image. This is called a supervised learning strategy, which aims to find an appropriate reconstruction function that matches the given image with the expected output. This can be expressed with the following equation:

(5) $$R|=|{\left\{(\mathrm{NN}\right)}_{L}{(F}^{–1}\left(\hat{y}))\right\}$$

where R is the reconstructed image, NN is a DL-based neural network, and L measures the loss between the original and reconstructed images.

**Figure 2:**
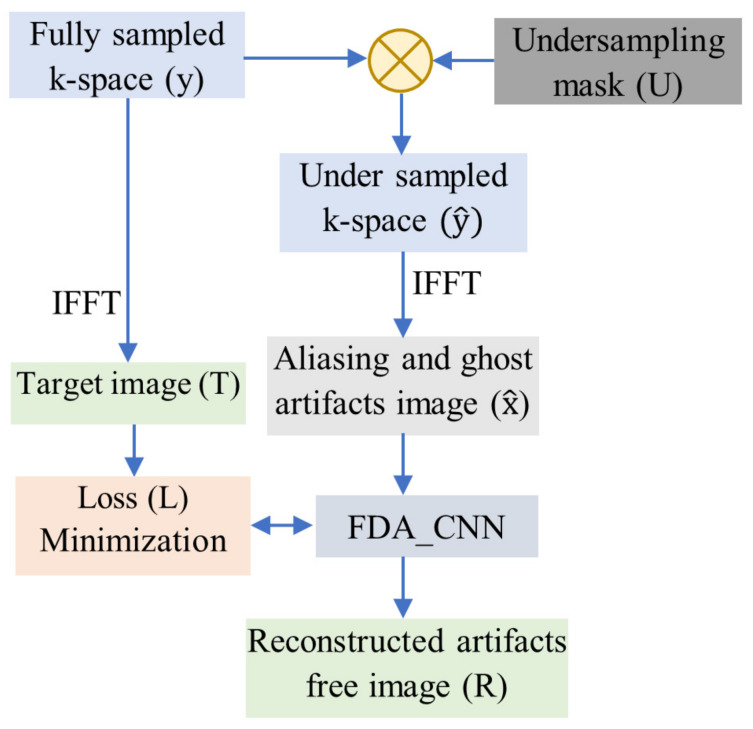
Flowchart of the proposed de-aliasing technique.

As shown in Figure 2, the fully sampled k-space (y) is sub-sampled by means of element-wise multiplication with the under-sampling mask (U). First, an image with aliasing and ghost artifacts $\hat{x}$ is reconstructed using the inverse fast Fourier transform (IFFT) from sub-sampled k-space $\hat{y}$. Then, these artifacts are removed by FDA-CNN by decreasing the loss (L) between the predicted image (R) and the target image (T). FDA-CNN attempts to recover an image $F^{-1}\left(\hat{y}\right)$ that is close to the target image using sub-sampled sensor data $\hat{y}$ as input. Gradient descent is used to optimize the parameters of the loss function.

### 2.2. Proposed FDA-CNN Architecture

The proposed MRI de-aliasing network architecture is presented in this section. We designed an improved CNN model based on the Unet [45] architecture by combining modified dense connectivity and AGs. We applied batch normalization [46] to accelerate training compared with earlier Unet implementations.

**Figure 3:**
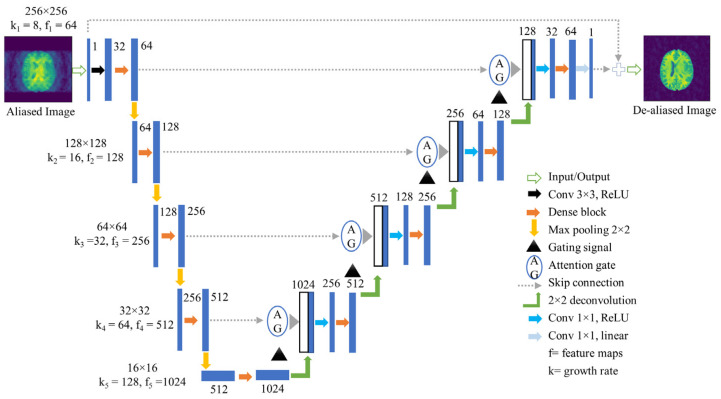
Proposed FDA-CNN architecture.

The network structure shown in Figure 3 has two main sections: the down- and up-sampling parts, and the skip connection part with the AGs. The down-sampling section consists of convolution, dense block, and max-pooling layers, whereas up-sampling consists of upscaling (2 × 2 deconvolution), dense block, and convolutional layers. Firstly, a 1-channel 3 × 3 convolutional operation is applied on a 256 × 256 input image with rectified linear unit (ReLU) [47] activation function. Then, five consecutive dense blocks (DB) are used. The first DB starts with 32 channels and gradually increases by 64, 128, 256, and 512. Every DB consists of a series of 1 × 1 and 3 × 3 convolutions with padding 1, batch normalization, and ReLU. Initially, hyperparameters $k_{m}$ and $f_{m}$ are specified by the user; for our method, we initialized $k_{1}=8$ and $f_{1}$ = 64. Then, $k_{m}$ and $f_{m}$ are changed by $k_{m}{=2}^{m-1}{\times \;k}_{1}$ and $f_{m}{=2}^{m-1}{\times \;f}_{1}$, respectively. The concatenation of the inputs and outputs from every layer of the DB generates its final output. Except for the last DB of the encoding section, a max-pooling operation is executed after each DB, which halves the size of the input at each level and doubles the number of feature maps.

The decoding or up-sampling section restores the size of the feature maps and maintains a form symmetric to the encoding section. This symmetry enables the reuse of features by concatenating feature maps at the same level and reduces the loss of information caused by the encoding/decoding process. Before concatenation, the features of encoding and decoding layers go through the AG to focus on target features from different spatial information. Every layer of the decoding section executes a 1 × 1 convolution with padding 1 and ReLU before going into a DB. A 1-channel 1 × 1 convolution is executed before generating the final output.

Moreover, dense connectivity generates deeper networks. For comparison, Unet has 23 layers, while FDA-CNN has 97 convolutional and deconvolutional levels. The vanishing gradient problem arises because the gradient information must flow through different layers and may disappear before it arrives at the succeeding layers. Dense connectivity adds more links to enable the effective backpropagation of gradient information. This lessens the vanishing gradient issue and makes it easier to train the network.

### 2.3. Dense Block

Densely connected networks [48] maximize the capability of the network by reusing features. The input of the succeeding layers is more varied and more effective when feature maps from various layers are combined. In our method, a dense block with a growth rate, $k_{m},$ is used to learn different feature maps, $f_{m,}$ for each spatial level, m. Initially, hyperparameters $k_{m}$ and $f_{m}$ are specified by the user. Then, $k_{m}$ and $f_{m}$ are changed by $k_{m}{=2}^{m-1}{\times \;k}_{1}$ and $f_{m}{=2}^{m-1}{\times \;f}_{1}$, respectively, at each spatial level to preserve computational efficiency and ensure that each dense block has the same number of convolutional layers. A total of nine dense blocks with four layers are used in the FDA-CNN approach.

**Figure 4:**
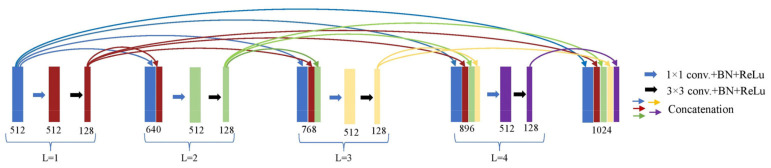
Last dense block with four layers of encoding part, where $k_{5}$ = 128 and $f_{5}$ = 512.

As shown in Figure 4, the $L^{\mathrm{th}}$ layer of the dense block has an initial input with F+× (L−1) feature maps and output with $k_{m}$ feature maps, where F is the total number of feature maps in the dense block’s initial input. Through a series of 1 × 1 and 3 × 3 convolutions with batch normalization plus ReLU activation function, features are learned. Due to the increased computational complexity of the 3 × 3 convolution, the input dimension is decreased to F feature maps by applying a 1 × 1 convolution, which increases convergence speed. Then, using a 3 × 3 convolution, $k_{m}$ attribute maps are developed from the compacted data. The concatenation of the inputs and outputs from every layer of the dense block generates the dense block’s final output.

### 2.4. Attention Gate

Models trained with AGs [49] intuitively learn to emphasize prominent features that are helpful for a particular task while suppressing irrelevant regions in an input image. With no additional computational work, AGs may be quickly added to common CNNs, such as Unet topologies, improving model sensitivity and prediction accuracy. Unet employs skip connections to merge spatial data from the up- and down-sampling paths. Low-quality feature representation exists in the first few layers, which carries in several redundant low-level feature extractions. By actively suppressing activations in unnecessary regions through the use of AGs at the skip connections, the number of redundant features transferred is decreased. Every AG takes two inputs, g and x. The gating signal, g, comes from the next lowest layer of the network. It has greater feature representation because it originates from a deeper region of the network. The input features, x, come from skipped connections. They have better spatial information because they originate from the early stages.

**Figure 5:**
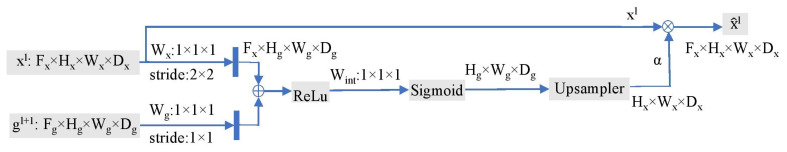
Schematic diagram of an attention gate.

We incorporate an AG with every decoding part in our fully dense Unet framework. As shown in Figure 5, input features $x_{i}^{l}$ perform 1 × 1 × 1 convolutions with stride 2 × 2 to lessen the size of the dimensions (H × W) by half, and gating signals $g_{i}^{l+1}$ perform 1 × 1 × 1 convolutions with stride 1 × 1. As a result, the spatial geometry of the modified input features and gating signals is the same. The ReLU function activates them through element-wise summation and maps them by $W_{\mathrm{int}}^{T}$ into a smaller-dimensional space for gating operations. The sigmoid function levels the vector in (0, 1), with coefficients closer to 1 denoting more pertinent features. Then, a trilinear up-sampler is used to restore the size of attention weight matrix $\alpha_{i}^{l}$ to correspond to the pixel density of the input features. The output of the AG, ${\hat{x}}_{i}^{l},$ is generated by means of element-wise multiplication between attention weight matrix $\alpha_{i}^{l}$ and input features $x_{i}^{l}$ and then is transmitted as usual through the residual connections.

## 3. Deep Learning Implementation

### 3.1. Datasets and Under-Sampling Masks

We used fully sampled brain k-space data from BraTs-2020 [50], fastMRI [51,52], and IXI [53]. We used the cross-sectional T1-weighted BraTS-2020 dataset for both the training and testing of all the networks. On the other hand, the T1-weighted axial fastMRI and T2-weighted coronal IXI datasets were only used for testing. Each volume possessed both the fully acquired k-space data and the associated reconstructed images of the same size (256 × 256). As we concentrated on the correlation between the number of k-space slices and FDA-CNN performance, no data augmentation was used in training. Images were reconstructed sequentially from every k-space.

During training, the sub-sampled zero-filled (ZF) noisy and artifact images were used as the network input along with the fully sampled images as target images. Our new MCP-US pattern was used for training and compared with 2D Gaussian under-sampling (2DG-US) and 1D Gaussian under-sampling (1DG-US) distributions. Mostly, 2D-US and 1DG-US focus on the central low frequencies of the k-space. However, low-spatial-frequency data, which determine the overall contrast, brightness, and form of the image, are located in the center of the k-space. On the other hand, high-spatial-frequency data determining the image edges and details are located in the periphery of the k-space. As the k-space has a symmetric nature, our MCP-US takes both the low and high frequencies. Among the total sampled data $\left(S\right)$ of each k-space, MCP-US constantly samples 50% of the center ${(s}_{c})$ and 25% of the periphery ${(s}_{p})$ and randomly chooses 25% of data ${(s}_{r})$ frequencies, except for center and periphery data. It can be expressed as follows:

$${S=s}_{c}{+s}_{p}{+s}_{r}$$

$${\mathrm{where}\;s}_{c}=\frac{S}{2}{\%,\;s}_{p}=\frac{S}{4}{\%,\;s}_{r}=\frac{S}{4}\%$$

**Figure 6:**
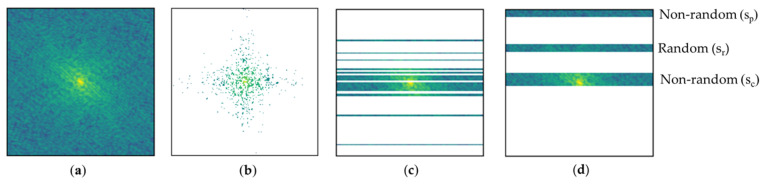
Several sampling patterns: (**a**) fully sampled; (**b**) 2D Gaussian pattern; (**c**) 1D Gaussian pattern; (**d**) mixed center and periphery under-sampling pattern.

In both training and testing, we took only 20% of data of each k-space for all three sub-sampling patterns, where white spaces were replaced with zero, as shown in Figure 6.

Among the sampled data $\left(S\right)$, our MCP-US non-randomly takes 10% of $s_{c}$ from the middle position and 5% of $s_{p}$ from the zero position and randomly chooses 5% of ${\;s}_{r}$ between the $s_{c}$ and $s_{p}$ areas. As we concentrated on the correlation between the number of training images and FDA-CNN performance, no data augmentation was used in training.

### 3.2. Loss Function

The disparity between the sub-sampled aliasing image and the fully sampled aliasing-free image was evaluated using the loss function. The optimum objective of FDA-CNN is to minimize the value of the loss function. Smaller values between the under-sampled and fully sampled images ensure better reconstruction. We used the mean square error (MSE) as the loss function to calculate pixel-wise disparity and update the network parameters, which can be expressed as follows:

(6) $${\mathrm{Loss}}_{\mathrm{MSE}}|=|\frac{1}{N}\sum_{i|=|1}^{N}{{(T}_{i}{–R}_{i})}^{2}$$

where N indicates the number of voxels (or pixels) in the image, and $T_{i}$ and $R_{i}$ represent the target and reconstructed MR images, respectively.

### 3.3. Performance Evaluations Metrics

To evaluate the network performance, we summarized the findings using four parameters: structural similarity index measure (SSIM) [54], peak signal-to-noise ratio (PSNR), normalized root mean squared error (NRMSE), and pixel visual information fidelity (VIFP) [55]. The SSIM is a perceptual index that utilizes the mutual dependencies among adjacent pixels to measure the similarity of two images, such as brightness, contrast, and structural properties. The following expression gives the SSIM between the network output (R) and the desired output (T):

(7) $$\mathrm{SSIM}\;\left(T,\;R\right)|=|\frac{\left({2\mu }_{T}\mu_{R}{+c}_{1}\right)\left({2\sigma }_{\mathrm{TR}}{+c}_{1}\right)}{\left(\mu_{T}^{2}{+\mu }_{R}^{2}{+c}_{1}\right)\left(\sigma_{T}^{2}{+\sigma }_{R}^{2}{+c}_{1}\right)}\;$$

where $\mu_{T}$ and $\mu_{R}$ represent the mean values of T and R, respectively; and $\sigma_{T}^{2}$ and $\sigma_{R}^{2}$ denote the corresponding pixel variance values. The covariance value is also shown by $\sigma_{\mathrm{TR}}$. To stabilize the division, $c_{1}$ and $c_{2}$ have the following definitions:

$$c_{1}|=|{(0.01P)}^{2}{,\;c}_{2}|=|{(0.03P)}^{2}$$

where $P|=|\max\left(T\right)–\min\left(T\right)$

The PSNR calculates the ratio of the signal’s highest potential power (image intensity throughout a volume) to its fidelity-affecting distorting noise power. This can be expressed as

(8) $$\mathrm{PSNR}\left(T,\;R\right)|=|{10\log}_{10}\left(\frac{255}{\sqrt{\frac{1}{N}\sum_{i|=|1}^{N}{{(T}_{i}{–R}_{i})}^{2}}}\right)\;$$

The ground truth and the pixel differences in network output images are compared by the NRMSE, which can be expressed as

(9) $$\mathrm{NRMSE}\left(T,\;R\right)|=|\frac{\sqrt{\frac{1}{N}\sum_{i|=|1}^{N}{{(T}_{i}{–R}_{i})}^{2}}}{\max\left(T\right)–\min\left(R\right)}$$

The human viewer’s perceptual evaluation approach, VIFP, measures image information by computing two mutual information quantities from the reference and distorted images. This can be defined as

(10) $$\mathrm{VIFP}\left(T,R\right)|=|\frac{\sum_{j\in \mathrm{subbands}}I\left(C^{\to N,j}{;\;R}^{\to N,j}{|s}^{N,j}\right)}{\sum_{j\in \mathrm{subbands}}I\left(C^{\to N,j}{;\;T}^{\to N,j}{|s}^{N,j}\right)}$$

where $R^{\to N,j}$, and $T^{\to N,j}\;$ represent the sub-bands of the reconstructed and target images, respectively; $S^{N,j}$ defines a realization for a specific image; and $C^{\to N,j}\;$ expresses N elements of random field $C_{j}$ that specifies the coefficient of the sub-band, j. The evaluation result of VIFP is indicated as values between 0 and 1, similar to the SSIM.

These criteria were chosen as they are typically used to evaluate image reconstruction. Higher values of SSIM, PSNR, and VIFP indicate better results, while smaller values of the NRMSE define better reconstructions. Moreover, the reconstruction time for each image indicates the transformation of MRI raw data into pictures. The reconstruction time of each method was calculated using the MCP-US pattern.

### 3.4. Experimental Setup

**Figure 7:**
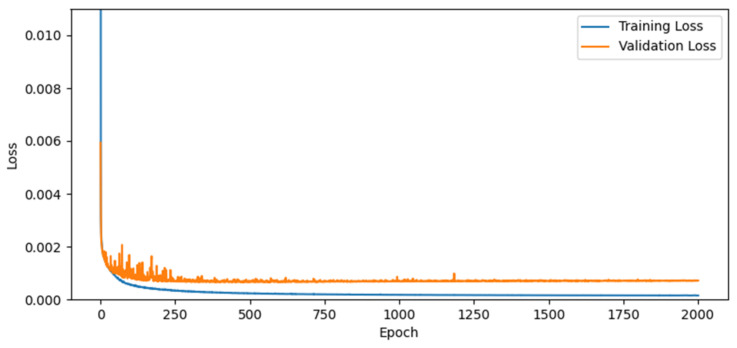
Training and validation losses of proposed FDA-CNN.

The training and testing of FDA-CNN were executed on an Intel Core i7-9800X 3.80 GHz processor and 128 GB memory with NVIDIA GeForce RTX 2080 Ti GPU running on Windows 10 Pro 64 bit. This model was implemented in Python 3.8 with the DL open-source libraries TensorFlow v2.4 and Keras v2.4 in the PyCharm environment. We used the Adam optimizer with momentum values of β1 = 0.9 and β2 = 0.999 to reduce the loss function. The starting learning rate was ${1\;\times \;\mathrm{10}}^{-4}$, and it declined with a decay factor of 0.95 every 20 epochs. A small batch size of 8 was chosen, and 2000 epochs were executed for the training of our network. Figure 7 illustrated the training and validation losses of the proposed network, where the regularizing impact of extensive connectivity lessens the possibility of overfitting the training data.

## 4. Result and Discussion

The performance of our network was compared to classical CS total variation (TV) [56], wavelet [57] denoising algorithms, and DL-based state-of-the-art (image and dual domain) reconstruction methods. Lightweight autoencoder (LAE) [58], basic Unet [46], projection-based cascade Unet (PBCU) [59], and DRL-based MRI (DRL-net) [41] reconstructions are image-domain DL networks. LAE uses an autoencoder framework, and PBCU uses five cascade Unets for MRI reconstruction. DRL-net subtracts the predicted artifacts from the under-sampled aliased images. The multidomain MRI reconstruction strategy (Wnet) [60] uses two Unets: one for the k-domain and another for the image domain. SSIM, PSNR, and NRMSE were used for quantitative analysis, where VIFP was used to evaluate the perception of the de-aliased images of a human viewer. The average reconstruction times were calculated using the MCP-US pattern. All results were generated in the same environment.

### 4.1. BraTs 2020-T1 Dataset

The T1-weighted axial brain Brats-2020 dataset was used for both the training and testing of all the networks. In an ideal dataset, the training and test data are very well correlated, providing an opportunity to acquire, from the training data, most of the features that are required to perform effectively during testing. The efficiency of CNNs can be compared in this ideal situation without being affected by the data. This BraTS-2020 dataset was obtained using a clinical 3T multimodal MR scanner. Among 150 k-spaces, 100 k-spaces were used for training; 30 k-spaces were used for validation; and 20 k-spaces were used for testing. Each k-space contained 155 axial cross-sectional T1-weighted (256 × 256) images. In the training, validation, and test sets, there was no duplication of the same k-space.

In this experiment, the same MRI sequence was used for both training and testing, with 25% of the training data being used for validation to increase the reliability of the results. The learning potential of FDA-CNN to eliminate artifacts was measured by adjusting the hyperparameters (feature maps and growth rate) of the dense block. Comparative DL methods were trained using the MCP-US pattern and evaluated on the associated datasets with each under-sampling mask. The efficiencies of all CNNs in terms of eliminating artifacts were compared using various sub-sampling masks for an acceleration factor of 5. In general, the CNN produced a better image with minimal artifacts. As seen in Table 1, the proposed FDA-CNN produced higher average SSIM, PSNR, and VIFP with a lower average NRMSE than traditional CS and the autoencoder and Unet-based methods.

The classical CS methods required almost 0.5 s and 0.97 s in reconstruction time for each slice, and the dual-domain network required approximately 0.4 s. In contrast, the single-domain post-processing methods required approximately 0.30 s to 0.33 s. Our proposed method generated better images than Unet within the same reconstruction time of 0.30 s for each slice.

**Figure 8:**
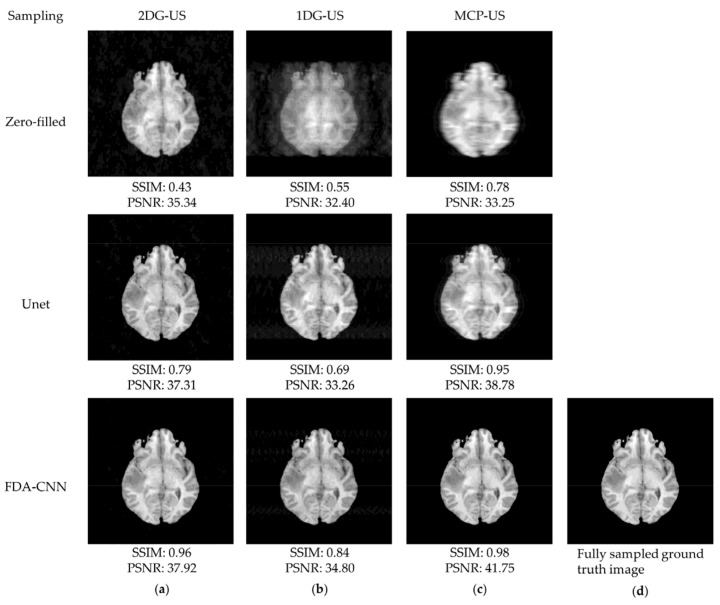
Reconstructed MRI images from the BraTs testing dataset (slice No. 140) using zero filling, Unet, and FDA-CNN: (**a**) 2D Gaussian distribution; (**b**) 1D Gaussian distribution; (**c**) mixed center and peripheral mask; (**d**) fully sampled ground truth image.

**Figure 9:**
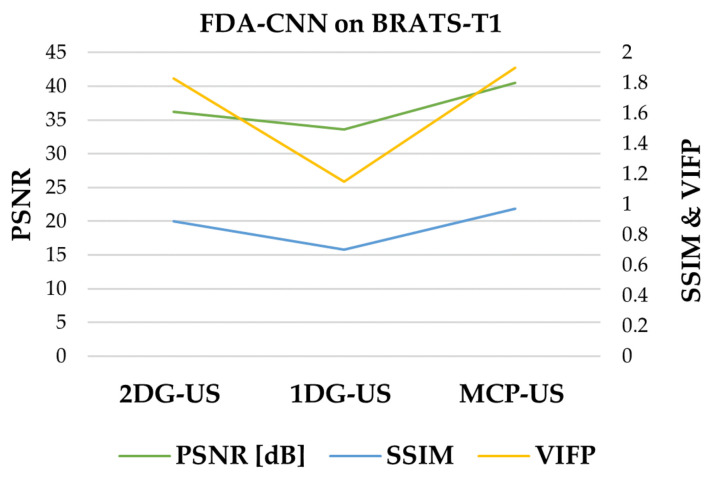
Comparison of three sampling patterns using FDA-CNN.

As shown in Figure 8, the under-sampled image contained noise and aliasing artifacts. Basic Unet improved the image quality by reducing these artifacts, although some artifacts remained. Our method effectively removed most of the artifacts and reconstructed the images close to the reference images from the BraTs testing dataset. The performance of three under-sampling masks on BraTs testing data is shown in Figure 9, where MCP-US generated better PSNR and SSIM than other under-sampling patterns, except for VIFP using 2DG-US.

### 4.2. FastMRI and IXI Datasets

The second experiment used the fastMRI and IXI datasets to test the CNNs after they had been trained on the BraTs dataset. This represents a scenario using different training and test datasets that are not perfectly matched. For testing, fastMRI comprised 2560 T1-weighted axial brain images from 160 k-spaces, while IXI comprised the same number of T2-weighed coronal brain images from 10 k-spaces. BraTs and fastMRI have many similarities, such as features and MR sequences. On the other hand, BraTs and IXI have distinctly dissimilar features and are not compatible due to their MR sequences. This experiment was performed to assess how well the CNN performs and generalizes when the training and testing datasets are different. The results of FDA-CNN and other methods without fine-tuning the fastMRI dataset are shown in Table 2.

**Figure 10:**
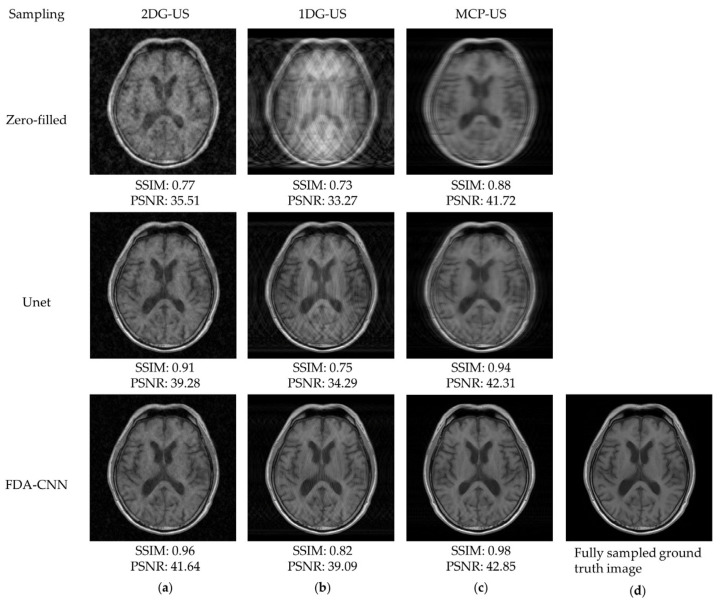
Reconstructed MRI images from the fastMRI dataset (slice No. 01) using zero filling, Unet, and FDA-CNN: (**a**) 2D Gaussian distribution; (**b**) 1D Gaussian distribution; (**c**) mixed center and peripheral mask; (**d**) fully sampled ground truth image.

**Figure 11:**
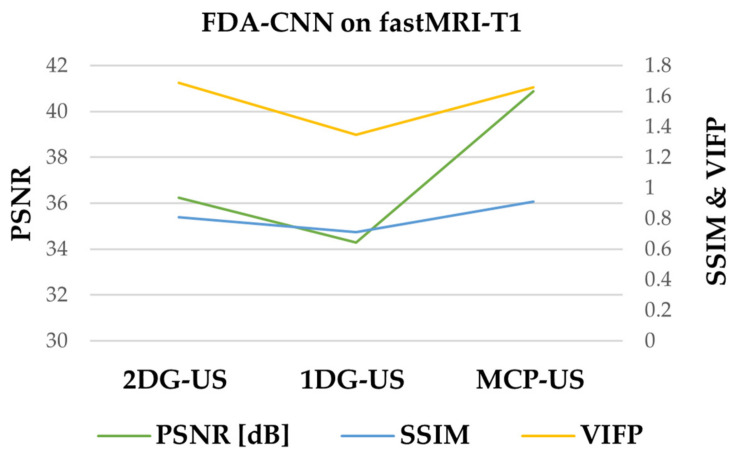
Comparison of three sampling patterns on fastMRI dataset using FDA-CNN.

With the 2DG-US mask, the CS methods slightly improved image quality, but under the other two Cartesian samplings, these methods did not perform well. The CNNs removed the artifacts and improved the image quality using all three sub-sampling patterns. Instead of VIFP, our method produced better average PSNR, SSIM, and NRMSE than the multidomain (Wnet) network in two cases. As shown in Figure 10, FDA-CNN effectively removed most of the artifacts and generated a better image than Unet. The performances of three under-sampling masks on the fastMRI dataset are shown in Figure 11. MCP-US produced better PSNR and SSIM than other under-sampling patterns, except for VIFP using the 2D random sampling pattern.

The test results of FDA-CNN and other methods on the IXI dataset are shown in Table 3. In this case, the CS methods improved some quantitative values but decreased the VIFP values. FDA-CNN performed significantly better and yielded high-quality images by eliminating unwanted artifacts compared with other networks using all masks. However, Wnet generated better VIFP in two Cartesian samplings than our method. The goal of this experiment was to determine whether it is feasible to test CNNs on unknown testing datasets to remove artifacts from anatomically accurate MR images using various sampling patterns.

**Figure 12:**
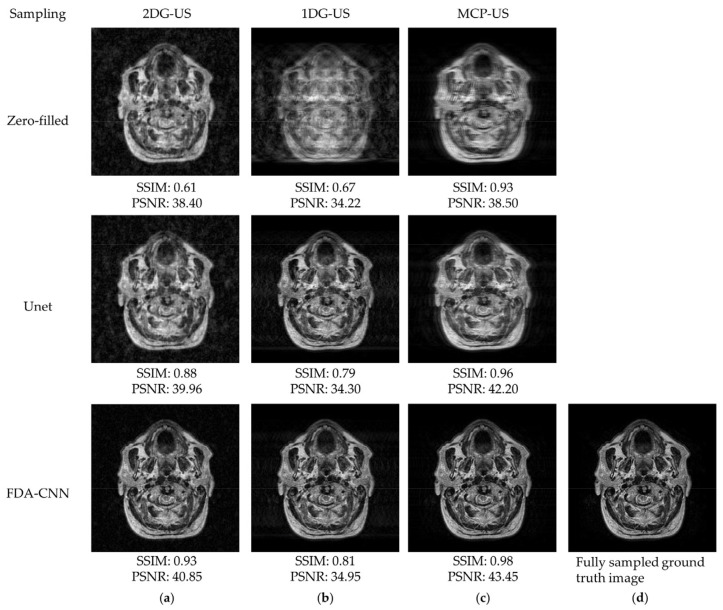
Reconstructed MRI images from IXI dataset (slice No. 75) using zero filling, Unet, and FDA-CNN at a sampling rate of 20%: (**a**) 2D Gaussian distribution; (**b**) 1D Gaussian distribution; (**c**) mixed center and peripheral under-sampling; (**d**) fully sampled ground truth image.

**Figure 13:**
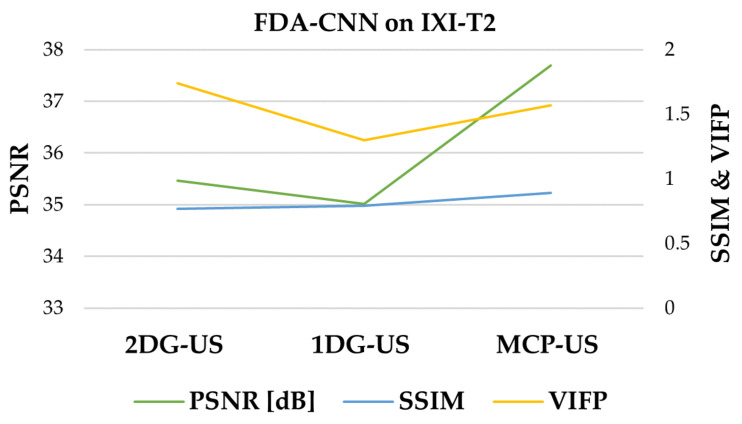
Comparison of three sampling patterns on IXI dataset using FDA-CNN.

As shown in Figure 12, FDA-CNN effectively reconstructed a better image, which was close to the reference image from different MRI sequence data, than Unet. The performances of three under-sampling patterns on the IXI dataset are shown in Figure 13. MCP-US performed better than other under-sampling patterns, except for VIFP using 2DG-US.

**Figure 14:**
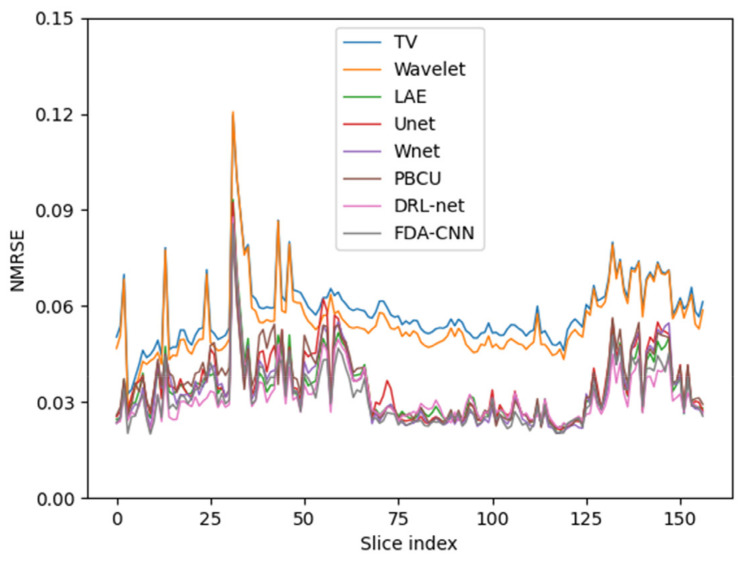
Average NRMSE variation across the slices. Edge slices have larger errors.

The average NRMSEs of the BraTs test dataset slices are displayed in Figure 14. The NRMSEs exhibited a recognizable pattern over the middle slices. The borders of the brain contain a lower number of frequencies that produce more unspecified and inconsistent images.

FDA-CNN performed better in artifact removal and image restoration than the regular Unet-based CNN and CS techniques according to the described test results. The two 3 × 3 convolutions in Unet are replaced by a dense block in FD-CNN. The input and output of all of the convolutional layers are comparatively small, although the dense block has eight distinct convolutional layers (four 1 × 1 and four 3 × 3). Therefore, the computational cost of the dense block-based convolutional layer is less expensive than that of Unet. Additionally, the regularizing impact of extensive connectivity lessens the possibility of overfitting the training data. The effectiveness of the CNN is heavily reliant on the accuracy of the MRI spatial frequencies, which is a drawback of post-processing techniques such as FDA-CNN. CNN reconstruction is likely to restore image features inaccurately if they are heavily obscured. Some of the lower frequencies may be recoverable if the CNN is directly employed to restore the sensor data. Furthermore, FD-CNN is more generalized than other state-of-the-art methods, as it generated higher average SSIM and PSNR, and lower average NRMSE on both the fastMRI and IXI datasets.

## 5. Conclusions

This article presents an efficient and effective deep learning-based method for MRI reconstruction from a sparsely sampled k-space using a fully dense attention convolutional neural network. In the proposed approach, edge information and geometry structure are restored more effectively from zero-filled MRI images. This network has the competency to extract realistic features and reconstruct 2D images that are virtually similar to the original. Dense connectivity remarkably promotes feature reuse and improves information flow within the network. Furthermore, AGs combine lower and higher spatial information to pick up more useful features, so the model needs a smaller number of parameters than the more complex Unet. This makes the network more generalized. Although network training requires many hours, reconstruction can be performed fast after training. Compared with CS-based iterative existing approaches, the proposed network needs less reconstruction time.

Compared with existing DL-based denoising and de-aliasing methods, the proposed network shows outstanding performance with regard to quantitative and qualitative human vision indexes, and reconstruction time. Furthermore, the correlations between the acquired image quality and several under-sampling patterns were evaluated. Future research will focus on recovering unmeasured frequencies in the k-domain. Moreover, we will implement our approach for real-time interactive temperature-based MRI.

## Figures and Tables

**Table 1:** Average SSIM, PSNR, NRMSE, and VIFP values and reconstruction time of state-of-art methods on BraTs-T1 axial brain data using several sampling patterns.

Sampling	Metrics	Zero Filling	TV	Wavelet	LAE	Unet	Wnet	PBCU	DRL-Net	FDA-CNN
2DG-US	SSIM	0.31	0.32	0.36	0.78	0.79	0.77	0.82	0.83	0.89
	PSNR	34.11	35.90	36.13	35.16	35.31	35.29	35.55	35.84	36.22
	NRMSE	0.09	0.07	0.06	0.08	0.08	0.07	0.07	0.06	0.04
	VIFP	0.60	0.50	0.65	0.87	0.92	0.93	0.90	0.80	0.94
1DG-US	SSIM	0.52	0.58	0.62	0.68	0.69	0.68	0.69	0.69	0.70
	PSNR	33.20	33.23	33.25	33.23	33.26	33.10	33.15	33.40	33.59
	NRMSE	0.12	0.12	0.12	0.12	0.12	0.12	0.12	0.11	0.10
	VIFP	0.25	0.21	0.27	0.42	0.38	0.44	0.38	0.41	0.45
MCP-US	SSIM	0.76	0.77	0.76	0.96	0.95	0.96	0.96	0.95	0.97
	PSNR	36.70	36.47	36.73	38.92	38.78	38.97	38.74	38.97	40.55
	NRMSE	0.05	0.06	0.06	0.03	0.04	0.03	0.04	0.03	0.02
	VIFP	0.52	0.41	0.50	0.90	0.91	0.91	0.89	0.85	0.93
Reconstruction time (seconds)			0.97	0.5	0.30	0.30	0.39	0.33	0.31	0.30

**Table 2:** Average SSIM, PSNR, NRMSE, and VIFP values and reconstruction time of state-of-art methods on fastMRI-T1 axial brain data using several sampling patterns.

Sampling	Metrics	Zero Filling	TV	Wavelet	LAE	Unet	Wnet	PBCU	DRL-Net	FDA-CNN
2DG-US	SSIM	0.52	0.49	0.51	0.70	0.72	0.72	0.76	0.80	0.81
	PSNR	35.87	35.65	35.89	35.16	35.28	35.13	35.51	35.40	36.23
	NRMSE	0.07	0.07	0.07	0.08	0.08	0.08	0.08	0.07	0.06
	VIFP	0.78	0.56	0.79	0.84	0.80	0.85	0.82	0.81	0.88
1DG-US	SSIM	0.57	0.54	0.57	0.61	0.64	0.63	0.65	0.66	0.71
	PSNR	32.64	32.62	32.64	32.89	33.08	32.71	33.03	33.18	34.29
	NRMSE	0.14	0.14	0.14	0.13	0.13	0.14	0.13	0.11	0.09
	VIFP	0.43	0.29	0.42	0.64	0.62	0.69	0.64	0.56	0.64
MCP-US	SSIM	0.78	0.76	0.78	0.88	0.87	0.88	0.88	0.88	0.91
	PSNR	36.76	36.40	36.76	37.79	37.93	37.88	37.76	37.81	40.88
	NRMSE	0.06	0.06	0.06	0.05	0.04	0.04	0.05	0.04	0.02
	VIFP	0.56	0.40	0.54	0.75	0.77	0.82	0.80	0.73	0.75
Reconstruction time (seconds)			1.52	1.3	0.46	0.49	0.95	0.82	0.47	0.44

**Table 3:** Average SSIM, PSNR, NRMSE, and VIFP values and reconstruction time of state-of-the-art methods on the IXI -T2 coronal dataset using several sampling patterns.

Sampling	Metrics	Zero Filling	TV	Wavelet	LAE	Unet	Wnet	PBCU	DRL-Net	FDA-CNN
2DG-US	SSIM	0.46	0.48	0.50	0.67	0.70	0.71	0.73	0.76	0.77
	PSNR	33.97	33.46	33.98	34.25	34.85	34.71	35.02	34.75	35.47
	NRMSE	0.15	0.07	0.07	0.10	0.08	0.08	0.08	0.07	0.06
	VIFP	0.69	0.70	0.67	0.89	0.86	0.90	0.86	0.75	0.97
1DG-US	SSIM	0.50	0.52	0.56	0.64	0.66	0.66	0.68	0.71	0.79
	PSNR	33.38	33.47	33.67	33.43	33.63	33.34	33.64	34.14	35.02
	NRMSE	0.12	0.12	0.12	0.12	0.11	0.12	0.11	0.09	0.07
	VIFP	0.35	0.19	0.32	0.58	0.57	0.60	0.55	0.56	0.51
MCP-US	SSIM	0.70	0.72	0.72	0.82	0.81	0.82	0.83	0.83	0.89
	PSNR	35.74	35.40	35.40	36.36	36.58	36.44	36.52	36.65	37.70
	NRMSE	0.07	0.08	0.08	0.06	0.06	0.06	0.06	0.05	0.04
	VIFP	0.44	0.25	0.25	0.71	0.73	0.77	0.73	0.68	0.68
Reconstruction time (seconds)			0.9	0.91	0.33	0.33	0.43	0.33	0.32	0.32

## Data Availability

The datasets are available at https://www.med.upenn.edu/cbica/brats2020/registration.html, https://fastmri.med.nyu.edu/ and https://brain-development.org/ixi-dataset/. The source code of this manuscript is available at https://github.com/biddut2j8/FDA-CNN (Last accessed on 20 December 2022).

## Abbreviations

1D	One-dimensional
1DG-US	1D Gaussian under-sampling
2D	Two-dimensional
2DG-US	2D Gaussian under-sampling
AG	Attention gate
CNN	Convolutional neural network
CS	Compressed sensing
CT	Computed tomography
DB	Dense block
DC	Dense connectivity
DL	Deep learning
DRL	Deep residual learning
FDA-CNN	Fully dense attention CNN
FFT	Fast Fourier transform
GAN	Generative adversarial network
IFFT	Inverse fast Fourier transform
LAE	Lightweight autoencoder
MCP-US	Mixed center and periphery under-sampling
MRI	Magnetic resonance imaging
MSE	Mean square error
NRMSE	Normalized root mean squared error
PAT	Photoacoustic tomography
PBCU	Projection-based cascade Unet
PI	Parallel imaging
PSNR	Peak signal-to-noise ratio
ReLU	Rectified linear unit
SSIM	Structural similarity index measure
TV	Total variation
VIFP	Pixel visual information fidelity
